# Transition from oxcarbazepine to eslicarbazepine acetate: A single center study

**DOI:** 10.1002/brb3.634

**Published:** 2017-01-27

**Authors:** Jussi Mäkinen, Sirpa Rainesalo, Jukka Peltola

**Affiliations:** ^1^ Department of NeurologyTampere University HospitalTampereFinland; ^2^ Department of NeurologyUniversity of Tampere and Tampere University HospitalTampereFinland

**Keywords:** epilepsy, eslicarbazepine acetate, oxcarbazepine, tolerability, treatment transition

## Abstract

**Objectives:**

There is limited clinical evidence for comparison between oxcarbazepine (OXC) and eslicarbazepine acetate (ESL) in terms of tolerability, or how to execute the change from OXC to ESL. We report the process of transitioning patients with focal epilepsy from previous OXC treatment to ESL due to tolerability problems. The rationale for change from OXC is reported, and the outcome with respective to this rationale is analyzed in terms of tolerability and efficacy.

**Materials and Methods:**

The subjects were transitioned overnight from OXC to ESL in a hospital inpatient setting. An evaluation of the effects of the transition was made after 1 and 3 months. All adverse events (AEs) were recorded following the transition period. Subjects were classified by outcome in terms of AEs.

**Results:**

Twenty‐three subjects were transitioned from OXC to ESL. Fifteen patients OXC‐related AEs reduced significantly after transition. Particularly, most of (93%) the AEs presented in the morning resolved after transition to ESL. No patient had an increase in seizure frequency following the transition. The incidence of ESL‐related AEs was 39% at 1 month and 13% at 3 month follow‐up; however, all patients continued ESL throughout the study period.

**Conclusions:**

This study demonstrates that patients suffering from OXC‐related AEs improve in terms of tolerability after a switch to ESL with maintaining seizure control. This improvement is more pronounced if the OXC‐related AEs are most evident following morning dosing of OXC. Transition can be safely executed in an outpatient setting.

## 1 Introduction

Epilepsy has an annual incidence of about 50 per 100,000 and prevalence between 5 and 10 per 1,000 (Sander, 2003). Monotherapy with an antiepileptic drug (AED) is sufficient to achieve seizure control without intolerable adverse events (AEs) approximately in 60% of patients (Stephen & Brodie, 2012). AEs related to AEDs impact negatively on health‐related quality of life, cause a significant source of disability, and may lead to low adherence to the treatment or treatment discontinuation (Stephen & Brodie, 2012).

In adults (>18 years), oral eslicarbazepine acetate (ESL) is approved in the EU as an adjunctive therapy with partial onset seizures with or without secondarily generalization and in the US as a monotherapy or adjunctive treatment of partial‐onset seizures (Aptiom®; Zebinix®). ESL is a third‐generation member of the dibenzazepine family, which also includes carbamazepine (CBZ) and oxcarbazepine (OXC; Keating, 2014; Zaccara, Giovanelli, Cincotta, & Verrotti, 2015). Blockade of voltage‐gated sodium channel (VGSC) is the proposed mechanism of action for CBZ, OXC, and ESL (Keating, 2014), but ESL has been shown to have a modulating action and inhibits the slow activation of VGSC (Hebeisen et al., 2015) and also an effect on Cav3.2T‐type Ca^2+^ channels (Doeser et al., 2015). ESL is a prodrug that is metabolized to its major active metabolite eslicarbazepine (S‐licarbazepine) and to the minor active metabolites (R)‐licarbazepine and OXC, which are mainly eliminated by renal excretion (both unchanged and glucuronide conjugate forms; Keating, 2014). Half‐life terminal elimination of ESL in plasma concentrations varies between 20–24 hr allowing once‐daily administration regimen (Perucca et al., 2011). Maximum plasma concentrations of ESL were reached in median 2.0–2.5 hr (Almeida & Soares‐Da‐silva, 2004). Steady state is reached in 4–5 days (Elger, Halász, Maia, Almeida, & Soares‐Da‐silva, 2009).

Eslicarbazepine acetate is efficacious and well tolerated as adjunctive therapy in drug‐resistant focal epilepsies at doses of 800 and 1,200 mg once‐daily (Ben‐Menachem et al., 2010; Elger et al., 2009; Gil‐Nagel, Lopes‐Lima, Almeida, Maia, & Soares‐Da‐silva, 2009; Gil‐Nagel et al., 2013; Sperling et al., 2015). Dizziness, vertigo, abnormal coordination, ataxia, diplopia, fatigue, somnolence, and headache are most often reported and frequent AEs in controlled clinical trials (Sperling et al., 2015) and to a lesser degree when used as the only adjunctive AED (Holtkamp, McMurray, Bagul, Sousa, & Kockelmann, 2016). It was noted in one study that switching from OXC to ESL (dose ratio 1:1) was associated with better tolerability during ESL treatment (Villanueva et al., 2014). Recent meta‐analysis compared the tolerability of ESL, OXC, and lacosamide (LCM) showing that patients with OXC withdrew from the treatment more frequently than patients with ESL or LCM (Zaccara, Giovanelli, Maratea, Fadda, & Verrotti, 2013). Furthermore, some side‐effects (diplopia, ataxia, abnormal coordination) were significantly more frequent in OXC‐treated patients compared to ESL and LCM. ESL‐related hyponatremia varies from 1.2% to 8.8% between different studies (Halasz et al., 2010; Hufnagel et al., 2013; Villanueva et al., 2014; Zaccara et al., 2013). The difference can be explained in terms of dosage used, population characteristics, and cut‐off used to define hyponatremia. These data suggest, that ESL might share similar efficacy compared to OXC, but with less AEs. Several dose‐dependent neurological AEs occur intermittently and appear almost always a few hours after OXC administration (Striano et al., 2006). It seems reasonable to relate AEs to OXC peak concentration rather than to the active metabolite eslicarbazepine, which levels increase more slowly (Keating, 2014). ESL is directly metabolized to eslicarbazepine with minor concentrations of r‐licarbazepine and OXC (Almeida & Soares‐Da‐silva, 2007).

At present, there is only one study documenting that overnight switch from OXC to ESL seemed to be safe and result in significant improvements in AEs, quality of life, and alertness (Schmid et al., 2016). However, that study focused on acute effects following the transition leaving long‐term effects of such transition related to tolerability and efficacy still unclear.

We provide 3 month follow‐up data when transitioning patients with focal epilepsy from previous OXC treatment to ESL in a standardized clinical setting. The rationale for change from OXC is reported, and 3 month outcome with respective to this rationale is analyzed in terms of tolerability (the main objective) and efficacy (the secondary objective).

## 2 Material and Methods

We identified all the patients (age at least 18 years) with focal epilepsy followed in the Department of Neurology in Tampere University Hospital, Finland from the patient registry. Following inclusion criteria were applied: (1) current treatment with OXC; (2) OXC‐related moderate or severe tolerability problems which affected daily life; (3) transition from OXC to ESL was performed due to OXC‐related AEs; and (4) transition was undertaken before 30 November 2015. The dosages of concomitant AEDs remained unchanged during the transition period. All subjects were on immediate‐release OXC as extended‐release OXC is not available in Finland. Information on the patient characteristics was obtained retrospectively from the medical records.

Tolerability problems related to OXC were categorized as in recent meta‐analysis (Zaccara et al., 2013) addressing neurological AEs of new generation sodium‐blockers (somnolence, dizziness, vertigo, ataxia/coordination abnormal, diplopia, nystagmus, fatigue, tremor, headache, nausea, vomiting). Patients were classified according to ILAE guidelines to temporal, frontal, parietal, occipital, multifocal, or unclassifiable epilepsies based on seizure characteristics, EEG and imaging findings and for some patients on ictal video‐EEG recordings (CoCaTotILA, 1989). The etiologies were divided into remote symptomatic and unknown. The seizure frequency from the previous year was recorded; seizure‐free patients did not have any seizures during the previous year. Refractory epilepsy was defined as having persistent seizures after trials of at least two AEDs with maximally tolerated doses (sequentially or in combination therapy).

Patients were transitioned overnight from OXC to ESL in a hospital inpatient setting on the day of arrival and the patients were followed up to 3 months by clinicians. The target dose of ESL was calculated using an OXC:ESL dose ratio 1:1 depending on the pretransition OXC dose. If the 1:1 dose ratio did not correspond to an exact ESL dose, then the closest lower ESL dose was used. The last intake of OXC was the morning dose followed by the first intake of ESL in the evening of the same day. After 1 and 3 months an evaluation of the effects of the transition was made in terms of tolerability, which was main purpose of this study. Efficacy in terms of seizure change was evaluated at 1 and 3 months after transitioning. All AEs and their intensity (mild, moderate, severe) were recorded and reported following the transitioning period. Mild was defined as a symptom not interfering with daily activities, moderate as interfering but not preventing daily life activities and severe as incapacitating at least part of daily activities. Patients were dichotomied by outcome in terms of AEs after switched from OXC to ESL.

This was a noninvasive, retrospective study, which does not oblige ethics committee approval according to Finnish Law on Research. Access to patient records based on decision made by Head of Science Centre, Tampere University Hospital research and innovation services, Science Center.

## 3 Results

**Figure 1:**
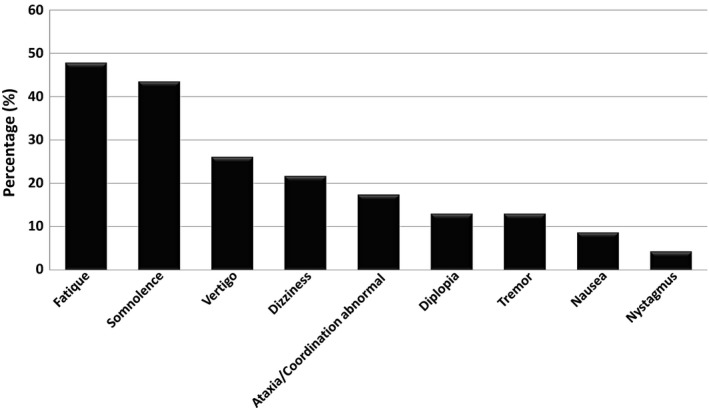
Adverse‐events related to oxcarbazepine. One or more adverse‐event can be present on a single subject

**Figure 2:**
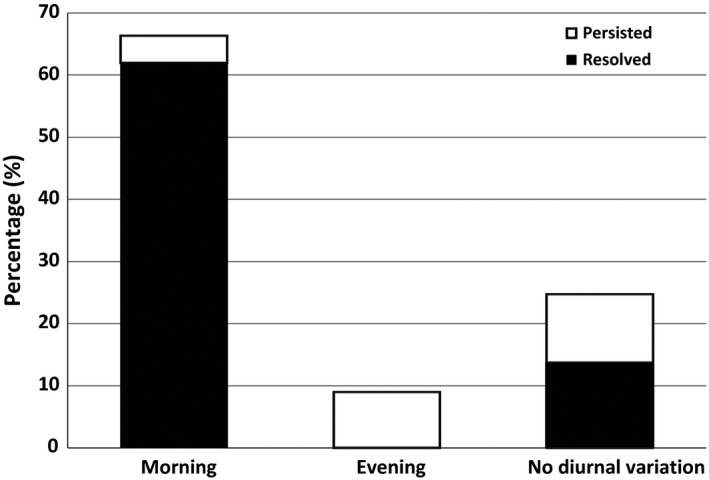
Diurnal variation and 3 month outcome of oxcarbazepine‐related adverse events after transition to eslicarbazepine acetate

We identified 23 patients, who were transitioned from OXC to ESL because of OXC‐related AEs. Demographic and medical characteristic of the subjects are presented in Table 1. Three most common concomitant AEDs were levetiracetam, topiramate, and clobazam. AEs related to OXC before transition to ESL are described in Figure 1. Fifteen (65.2%) patients OXC‐related AEs resolved after transition at 3 months follow‐up and this was the case in 14 patients at 1 month follow‐up. Furthermore, the timing of the AEs over the day with respect to their persistency was analyzed and the results are shown in Figure 2. Two thirds (66.5%) of the AEs occurred in the morning and most of them (93.4%) resolved after transitioning OXC to ESL. AEs presenting all day or evening tended to be more persistent after transition.

**Table 1:** Demographic and medical characteristics of the patients

Number of patients	23
Sex	
Female, *N* (%)	14 (60.9)
Male, *N* (%)	9 (39.1)
Mean age; years (range)	41.8 (22–69)
Mean duration of epilepsy; years (range)	14.4 (2–62)
Etiology	
Remote symptomatic, *N* (%)	17 (73.9)
Unknown, *N* (%)	6 (26.1)
Refractory epilepsy, *N* (%)	18 (78.2)
Seizure frequency	
Seizure free (during previous year), *N* (%)	11 (47.8)
Persistent seizures, *N* (%)	12 (52.2)
Mean OXC dose; mg/day (range)	1,152 (600–1,800)
Final ESL dose; mg/day(range)	1,095 (800–2,000)
Number of concomitant AEDs, *N* (%)	
0	3 (13.0)
1	9 (39.2)
2	10 (43.5)
3	1 (4.3)
Number of prior AEDs, *N* (%)	
1	3 (13.0)
2	5 (21.8)
3	6 (26.1)
4	3 (13.0)
≥5	6 (26.1)

AEDs, antiepileptic drugs; ESL, eslicarbazepine acetate; OXC, oxcarbazepine.

The most intolerable single OXC‐related AEs were somnolence, dizziness, and diplopia. Fatique, somnolence, tremor, diplopia, and nausea mostly resolved after transition. A clear tendency that some AEs would have been more persistent than others, especially in the morning, was not observed. However, dizziness and coordination problems were the commonest AEs that persisted after transition.

**Figure 3:**
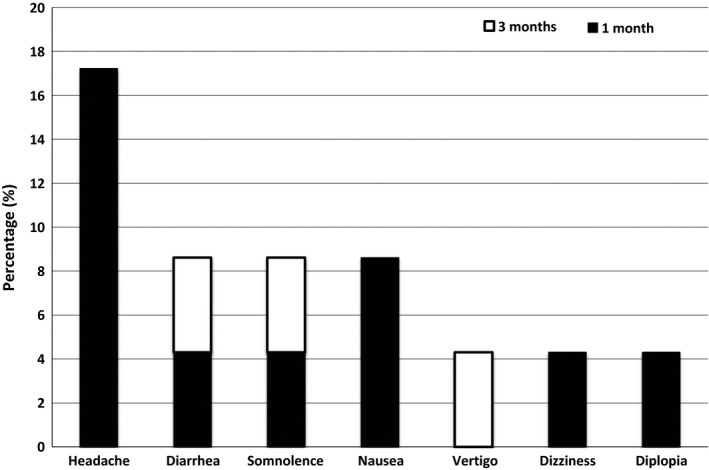
Duration of eslicarbazepine acetate‐related adverse events

The effects of the transition from OXC to ESL on seizure frequency are summarized in Table 2. AEs occurring after transition related to ESL are shown in Figure 3. The incidence of AEs was higher at 1 month (39.1% [9/23]) than at 3 month follow‐up (13.0% [3/23]). However, no treatment discontinuations occurred and all patients continued ESL throughout the study period. Most of the AEs attributed to ESL were mild and some moderate in intensity. The dose‐dependent increase in AEs frequency was not noticed.

**Table 2:** The effect of transition from oxcarbazepine to eslicarbazepine acetate on seizure frequency in 3 month follow‐up

Patient	Baseline SF (previous month)	1st month SF	2nd month SF	3rd month SF	Outcome
1–11	Seizure free	No seizures	No seizures	No seizures	Still seizure free
12	1 SGS, 31 SPS, 4 CPS	30 SPS, 3 CPS	32 SPS, 4 CPS	31 SPS, 6 CPS	No significant change in SF
13	Infrequent seizures^a^	No seizures	No seizures	No seizures	No significant change in SF
14	1 SGS, 5 CPS	2 CPS	No seizures	1 CPS	50% reduction in seizure frequency
15	1 SPS, 2 CPS	2 SPS, 2 CPS	1 SPS, 3 CPS	1 SPS, 2 CPS	No significant change in SF
16	83 SPS, 8 CPS	84 SPS, 8 CPS	83 SPS, 8 CPS	83 SPS, 8 CPS	No change in SF
17	5 CPS	4 CPS	5 CPS	6 CPS	No change in SF, seizure duration shortened
18	1 CPS	No seizures	1 CPS	No seizures	30% reduction in seizure frequency
19	Infrequent seizures^b^	No seizures	No seizures	No seizures	No significant change in SF
20	5 SPS, 8 CPS	5 SPS, 8 CPS	8 SPS, 11 CPS	7 SPS, 8 CPS	No change in SF, CPS seizure duration shortened
21	1 SGS, 3 SPS, 1 CPS	1 SGS, 3 SPS, 3 CPS	2 SPS, 2 CPS	1 SGS, 3 SPS, 1 CPS	No significant change in SF
22	2 SGS, 7 CPS	3 SGS, 8 CPS	2 SGS, 11 CPS	3 SGS, 6 CPS	No significant change in SF
23	1 SGS, 4CPS	1 CPS	4 CPS	2 SGS, 4 CPS	No significant change in SF

CPS, complex partial seizure; SF, seizure frequency; SGS, secondary generalized tonic‐clonic seizure; SPS, simple partial seizure.

a 1 SGS during previous year.

b 1 CPS during previous year.

Changes in seizure frequency or duration were not observed during the transition period in a hospital inpatient setting. Two patients reported headache during the hospitalization, but at 1 and 3 month follow‐up this AE was not observed in these patients any more. None of those patients who had AEs during the follow‐up did report tolerability problems during the hospitalization.

## 4 Discussion

The main objective of this study transitioning patients with focal epilepsy from OXC to ESL due to typical OXC‐related AEs was to evaluate the tolerability during sufficiently long follow‐up period. Our study demonstrates that patient satisfaction improved significantly in terms of reduced AEs after switching from OXC to ESL in 65% of the subjects without increase in seizure frequency. This finding is similar to a previous study demonstrating that 15 of 26 patients who were transitioned from OXC to ESL due to OXC‐related AEs no longer had AEs after the change (Villanueva et al., 2014). Furthermore, we showed that if the OXC‐related AEs are most evident in the morning (following morning dosing), nearly all of them (93%) dissolved after transition to ESL indicating a relation to OXC peak cerebrospinal fluid and plasma concentration as suggested earlier (Keating, 2014). These findings might help clinician in everyday practice to assess whether patient's complaints of neurological AEs related to OXC, especially in complex situations; several AEDs, comorbidities (e.g. depression, sleeping problems) and, persistent seizures.

The secondary objective was to assess the efficacy of ESL. The results of this study indicated that when switched from OXC, ESL was effective and well tolerated during 3 months follow‐up. During previous year 12 patients had persistent seizures and after changing from OXC to ESL in one patient seizure frequency reduced by 50% and in another patient by 30%. Moreover, in two patients seizure duration shortened without change in seizure frequency. Altogether four patients of 12 achieved reduction in seizure frequency or duration. Nevertheless, the fact that none of the patients had increased seizure frequency is at least as important as seizure reduction in small proportion of the patients.

Earlier expert group′s opinion hypothesized there might be situations in which it may be reasonable to convert patients from OXC to ESL; most appropriately those who experience OXC‐related AEs or have poor compliance with twice‐daily OXC dosing (Peltola et al., 2015). Recent study by Schmid et al. (2016) demonstrated that overnight switching from OXC to ESL was safe and successful with regard to efficacy concerning the acute and immediate effects on tolerability and seizure issues. Other major finding is the absence of seizure related or other problems during the transition in any of our patients which give added value to the publication by Schmid et al. (2016). Furthermore, we did not find any specific concerns, why transition from OXC to ESL should be done necessarily in an inpatient setting as done earlier in our center. Transition from OXC to ESL can be safely done in an outpatient setting.

There were slight differences how the prompt switch from OXC to ESL was conducted in our center in comparison to the previous study by Schmid et al. (2016). In that study the last intake of OXC was the evening dose followed by first intake of ESL in the evening of next day, whereas in our center the last intake of OXC was the morning dose followed by the first intake of ESL already in the evening of the same day. There were no differences between these two studies on how the initiation of ESL was performed in terms of target dosing as both studies aspired to use a ratio of 1:1 of OXC and ESL.

Considering the limitations, this was retrospective uncontrolled follow‐up study and the relative number of our patient is not high. However, when considering the results and conclusions emerging from our study the number of patients is justified as its' present form. The main conclusion is that such OXC‐related neurological side‐effects that appear after ingestion of the morning dose of OXC disappear in the vast majority (over 90%) of the patients when substituted with ESL, whereas if these symptoms exist either after evening dose or without diurnal variation the substitution is less helpful. This association is so strong that the current number of patients is sufficient to provide the conclusion.

In conclusion, our findings support the notion that patients currently receiving OXC and experiencing intolerable AEs benefit from switching to ESL in order to maintain seizure control and improve AED tolerability. This is particularly true if these AEs are most evident following morning dosing. Our data also suggest that transition from OXC to ESL can be performed safely in an outpatient setting instead of overnight hospitalization for cost effectiveness and patient comfort.

## Conflict of Interest

Jussi Mäkinen has received support for travel congresses from Biogen‐Idec, Boehringer‐Ingelheim, Eisai, and Orion Pharma; received speaker honoraria from Boehringer‐Ingelheim; received research funding from Finnish Epilepsy Association; and participated in advisory board for Eisai. Sirpa Rainesalo has received speaker honoraria from FennoMedical, Orion Pharma, UCB and received support for travel to congresses from Abbvie and UCB. Jukka Peltola has participated in clinical trials for Eisai, UCB, and Bial; received research grants from Eisai, Medtronic, UCB, and Cyberonics; received speaker honoraria from Cyberonics, Eisai, Medtronic, Orion Pharma, and UCB; received support for travel congresses from Cyberonics, Eisai, Medtronic, and UCB; and participated in advisory boards for Cyberonics, Eisai, Medtronic, UCB, and Pfizer.
